# Supplementary material for: Deep learning approaches for quantitative and qualitative assessment of cervical vertebral maturation staging systems
Source: PLoS One. 2025 May 20;20(5):e0323776. doi: 10.1371/journal.pone.0323776 (PMC12091812; doi:10.1371/journal.pone.0323776)
Supplement: S1 Table — (DOCX) [file pone.0323776.s001.docx]

| **Stage** | **Inferior Borders of C2, C3, C4** | **C3 Morphology** | **C4 Morphology** | **Timing intervention** |
| --- | --- | --- | --- | --- |
| CS 1 | Flat, Flat, Flat | Trapezoid | Trapezoid | Prepubertal stage |
| CS 2 | Concavity, Flat, Flat | Trapezoid | Trapezoid | Prepubertal ("get-ready") stage |
| CS 3 | Concavity, Concavity, Flat | Trapezoid | Trapezoid/Rectangular Horizontal | Circumpubertal stage |
| CS 4 | Concavity, Concavity, Concavity | Rectangular Horizontal | Rectangular Horizontal | Circumpubertal stage |
| CS 5 | Concavity, Concavity, Concavity | Square/Rectangular Horizontal | Square/Rectangular Horizontal | Post pubertal stage |
| CS 6 | Concavity, Concavity, Concavity | Rectangular Vertical/Rectangular Horizontal | Rectangular Vertical/Rectangular Horizontal | Post pubertal stage |

S1 Table of qualitative measurements of six stages and intervention
