# Supplementary material for: Deep learning approaches for quantitative and qualitative assessment of cervical vertebral maturation staging systems
Source: PLoS One. 2025 May 20;20(5):e0323776. doi: 10.1371/journal.pone.0323776 (PMC12091812; doi:10.1371/journal.pone.0323776)
Supplement: S2 Table — (DOCX) [file pone.0323776.s002.docx]

S2 Table The landmarks and definition for QCVM analysis.

| No. | Landmarks | Definition |
| --- | --- | --- |
| 1 | C2p | The most posterior point of C2 on the lower border |
| 2 | C2a | The most anterior point of C2 on the lower border |
| 3 | C2d | The deepest point of the concavity at the lower border of C2 |
| 4 | C3lp | The most posterior point of C3 on the lower border |
| 5 | C3up | The most posterior point of C3 on the upper border |
| 6 | C3ua | The most anterior point of C3 on the upper border |
| 7 | C3la | The most anterior point of C3 on the lower border |
| 8 | C3m | The deepest point of the concavity at the lower border of C3 |
| 9 | C4lp | The most posterior point of C4 on the lower border |
| 10 | C4up | The most posterior point of C4 on the upper border |
| 11 | C4ua | The most anterior point of C4 on the upper border |
| 12 | C4la | The most anterior point of C4 on the lower border |
| 13 | C4m | The deepest point of the concavity at the lower border of C4 |
