# Supplementary material for: Deep learning approaches for quantitative and qualitative assessment of cervical vertebral maturation staging systems
Source: PLoS One. 2025 May 20;20(5):e0323776. doi: 10.1371/journal.pone.0323776 (PMC12091812; doi:10.1371/journal.pone.0323776)
Supplement: S3 Table — (DOCX) [file pone.0323776.s003.docx]

S3 Table of quantitative measurements description

| No. | Measurements | Description |
| --- | --- | --- |
| 1 | C2Conc | Measure of the concavity depth at the lower border of C2 (distance from the line connecting C2p and C2a to the deepest point on the lower border of the vertebra, C2 m). |
| 2 | C3Conc | Measure of the concavity depth at the lower border of C3 (distance from the line connecting c3lp and c3la to the deepest point on the lower border of the vertebra, C3 m) |
| 3 | C4Conc | Measure of the concavity depth at the lower border of C4 (distance from the line connecting c4lp and c4la to the deepest point on the lower border of the vertebra, C4 m) |
| 4 | C3PAR (ratio) | The ratio between the posterior (distance c3up-c3lp) and anterior (distance c3ua-c3la) heights of the body of C3. |
| 5 | C3BAR (ratio) | The ratio between the length of the base (distance c3lp-c3la) and the anterior height (distance c3ua c3la) of the body of C3. |
| 6 | C4PAR (ratio) | Ratio between the posterior (distance c4up-c4lp) and anterior (distance c4ua-c4la) heights of the body of C4 |
| 7 | C4PAR (ratio) | The ratio between the length of the base (distance c4lp-c4la) and the anterior height (distance c4ua c4la) of the body of C4. |
